# Deciphering smooth muscle cell heterogeneity in atherosclerotic plaques and constructing model: a multi-omics approach with focus on KLF15/IGFBP4 axis

**DOI:** 10.1186/s12864-024-10379-y

**Published:** 2024-05-17

**Authors:** Zhanli Peng, Qinghui Kan, Kangjie Wang, Tang Deng, Shenming Wang, Ridong Wu, Chen Yao

**Affiliations:** 1 Division of Vascular Surgery, The First Affiliated Hospital, Sun Yat-sen University, No. 58 Zhongshan Er Road, Guangzhou, 510080 P.R. China; 2 National-Guangdong Joint Engineering Laboratory for Diagnosis and Treatment of Vascular Diseases, First Affiliated Hospital, Sun Yat-sen University, Guangzhou, P.R. China

**Keywords:** Atherosclerosis, Smooth muscle cell, Single-cell

## Abstract

**Background:**

Ruptured atherosclerotic plaques often precipitate severe ischemic events, such as stroke and myocardial infarction. Unraveling the intricate molecular mechanisms governing vascular smooth muscle cell (VSMC) behavior in plaque stabilization remains a formidable challenge.

**Methods:**

In this study, we leveraged single-cell and transcriptomic datasets from atherosclerotic plaques retrieved from the gene expression omnibus (GEO) database. Employing a combination of single-cell population differential analysis, weighted gene co-expression network analysis (WGCNA), and transcriptome differential analysis techniques, we identified specific genes steering the transformation of VSMCs in atherosclerotic plaques. Diagnostic models were developed and validated through gene intersection, utilizing the least absolute shrinkage and selection operator (LASSO) and random forest (RF) methods. Nomograms for plaque assessment were constructed. Tissue localization and expression validation were performed on specimens from animal models, utilizing immunofluorescence co-localization, western blot, and reverse-transcription quantitative-polymerase chain reaction (RT-qPCR). Various online databases were harnessed to predict transcription factors (TFs) and their interacting compounds, with determination of the cell-specific localization of TF expression using single-cell data.

**Results:**

Following rigorous quality control procedures, we obtained a total of 40,953 cells, with 6,261 representing VSMCs. The VSMC population was subsequently clustered into 5 distinct subpopulations. Analyzing inter-subpopulation cellular communication, we focused on the SMC2 and SMC5 subpopulations. Single-cell subpopulation and WGCNA analyses revealed significant module enrichments, notably in collagen-containing extracellular matrix and cell-substrate junctions. Insulin-like growth factor binding protein 4 (*IGFBP4*), apolipoprotein E *(APOE*), and cathepsin C (*CTSC*) were identified as potential diagnostic markers for early and advanced plaques. Notably, gene expression pattern analysis suggested that *IGFBP4* might serve as a protective gene, a hypothesis validated through tissue localization and expression analysis. Finally, we predicted TFs capable of binding to *IGFBP4*, with Krüppel-like family 15 (*KLF15*) emerging as a prominent candidate showing relative specificity within smooth muscle cells. Predictions about compounds associated with affecting *KLF15* expression were also made.

**Conclusion:**

Our study established a plaque diagnostic and assessment model and analyzed the molecular interaction mechanisms of smooth muscle cells within plaques. Further analysis revealed that the transcription factor *KLF15* may regulate the biological behaviors of smooth muscle cells through the *KLF15/IGFBP4* axis, thereby influencing the stability of advanced plaques via modulation of the PI3K-AKT signaling pathway. This could potentially serve as a target for plaque stability assessment and therapy, thus driving advancements in the management and treatment of atherosclerotic plaques.

**Supplementary Information:**

The online version contains supplementary material available at 10.1186/s12864-024-10379-y.

## Introduction

Atherosclerosis (AS), a leading pathological factor culminating in severe cardiovascular events such as stroke and myocardial infarction (MI), remains the primary global cause of mortality [1, 2]. Despite its gradual progression, the rupture of atherosclerotic plaques can result in substantial disability and fatalities [3]. Therefore, the stability of plaques is intricately linked to the occurrence of acute ischemic events [4]. Unstable plaques, characterized by their thin fibrous cap and larger necrotic cores [5], exhibit heightened susceptibility to cardiovascular events. Strategies aimed at preventing plaque progression toward instability or reversing instability to stability hold profound implications.

VSMCs, situated in the middle layer of the vascular wall, play a pivotal role in maintaining vascular tone and regulating blood pressure primarily through contraction [6]. In response to pathological factors, VSMCs undergo processes such as proliferation, dedifferentiation, and migration, which significantly influence the onset, development, and stability of plaques [7]. Phenotypic conversion towards fibroblast-like smooth muscle cells is particularly favorable for plaque stabilization [8] and has been observed in mouse plaque fibrous caps and human coronary plaques [9]. Additionally, studies have reported a correlation between the amount of VSMCs in the fibrous cap, as major producers of extracellular matrix (ECM) proteins, and plaque stability [10, 11]. Hence, alterations in the biological behavior of smooth muscle cells are crucial for plaque stability. While current therapeutic strategies for atherosclerosis predominantly focus on reducing low-density lipoprotein cholesterol [12]. Targeted gene therapy specific to VSMCs may represent a groundbreaking treatment approach.

Constrained by the limitations of RNA-seq in detecting and explaining interactions within cell populations, single-cell genomics has emerged as a valuable tool for dissecting cell population heterogeneity and tissue cell types within atherosclerotic plaques [9, 13]. Our study, based on single-cell and transcriptomic data from human atherosclerotic plaques, identified subpopulations of fibroblast-like smooth muscle cells and their closely interacting smooth muscle cell subpopulations. We delved into their biological functions, identified hub genes that may drive phenotypic transitions, and established a diagnostic assessment model. Among these genes, *IGFBP4*, distinguished as a protective gene in contrast to *APOE* and *CTSC*, displayed high expression levels in both normal and advanced plaques. Tissue-specific interventions hold significant promise. Building upon this, we further predicted TFs that could potentially bind to *IGFBP4* within smooth muscle cells. *KLF15*, recognized as a TF with relative specificity in smooth muscle cells, may interact with *IGFBP4*, potentially facilitating the phenotypic transition of smooth muscle cells into fibroblast-like smooth muscle cells. This offers novel perspectives for atherosclerosis prevention and the enhancement of plaque stability.

## Materials and methods

### Datasets used in this study

The datasets (GSE159677 [14], GSE28829 [15], GSE43292 [16], GSE100927 [17], and GSE20129 [18]) employed in this study were retrieved from the GEO database (http://www.ncbi.nlm.nih.gov/geo/) and are summarized in Table 1.

**Table 1:** Datasets information

GSE ID	Platform	Disease state1	Disease state2	Sample type	Purpose of dataset	Total
GSE159677	GPL18573	Calcified atherosclerotic core (AC) (*n* = 3)	Proximal adjacent (PA) (*n* = 3)	Plaque	Training set	6
GSE28829	GPL570	Early atherosclerotic plaque (*n* = 13)	Advanced atherosclerotic plaque (*n* = 16)	Plaque	Training set	29
GSE43292	GPL6244	Macroscopically intact tissue (stages I and II) (*n* = 32)	Atheroma plaque (stage IV and over of the Stary classfication) containing core and shoulders of the plaque (*n* = 32)	Plaque	Testing set	64
GSE100927	GPL17077	Healthy control (*n* = 69)	Atherosclerotic (*n* = 35)	Plaque	Testing set	104
GSE20129	GPL6104 GPL10558	Healthy control (*n* = 86)	Atherosclerotic (*n* = 49)	Blood	Testing set	135

### Single-cell data pre-processing

For the single-cell samples from GSE159677, we created a Seurat object using the “CreateSeuratObject” function within the Seurat R package (version 4.3.0.1) [19]. We retained cells that had more than 300 features and shared more than 3 genes. To assess mitochondrial genes, we utilized the “PercentageFeatureSet” function. Cells with less than 25% mitochondrial genes were included in the analysis.

### Batch effects removal, dimensionality reduction, and single-cell data clustering

The data underwent normalization through the “NormalizeData” function. Subsequently, we selected the top 2000 highly variable genes using the “FindVariableFeatures” function. Afterward, the data was scaled and subjected to principal component analysis (PCA). To eliminate batch effects, we employed the “harmony” package. Plaque cell clustering and VSMCs reclustering analysis were conducted by utilizing the “FindNeighbors” and “FindCluster” functions, with a resolution parameter set to 0.2. The t-Distributed Stochastic Neighborhood Embedding (t-SNE) was applied for data visualization following cell clustering. It’s worth noting that the “FindVariableFeatures,” “FindNeighbors,” and “FindCluster” functions are part of the Seurat package.

### Cell type annotation and analysis of differentially expressed genes

To annotate cell types, we integrated known cell markers and referred to the “Cell Taxonomy” database (https://ngdc.cncb.ac.cn/celltaxonomy/) [20]. We utilized the “FindAllMarkers” function with specified criteria (min.pct = 0.25, logfc.threshold = 0.25, *P* < 0.05) to identify differentially expressed genes (DEGs) for characterizing distinct cell subpopulations. Subsequently, we conducted a reclustering of smooth muscle cells and reannotated them into different subtypes based on smooth muscle cell markers and the “Cell Taxonomy” database.

### Analysis of cell-to-cell interactions

To analyze cell-to-cell interactions, we utilized the “CellChat” R package (Version 1.6.1) [21]. We followed the official protocol for extracting subpopulations of smooth muscle cells, allowing us to successfully create and normalize CellChat objects. The R package includes the “CellChatDB.human” databank, which we used to screen for receptor-ligand interactions. Subsequently, we quantified and visually represented potential ligand-receptor interactions, including their numbers and strengths, between cells. This was achieved using functions such as “computeCommunProb,” “computeCommunProbPathway,” and “aggregatNet” from the CellChat R package.

### Pseudotime trajectory analysis

For the analysis of pseudotime trajectories in a specific smooth muscle cell subpopulation, we employed the “Monocle2” R package (Version 2.26.0) [22, 23]. The construction of the pseudotime analysis object was carried out using the “newCellDataSet” function. Default parameters of the “reduceDimension” function were used for dimension reduction. Subsequently, we conducted cell trajectory analysis, gene dynamic expression analysis, and visualization using functions such as “plot_cell_trajectory” and “plot_genes_in_pseudotime.”

### Identification of DEGs in GSE28829

To identify DEGs within the GSE28829 dataset, we employed the “limma” package in R software [24]. Our analysis focused on two distinct groups, namely, ‘Early Atherosclerotic Plaque’ and ‘Advanced Atherosclerotic Plaque’, based on the following criteria: |logFC| > 0.75 and adjusted p-value < 0.05.

### WGCNA of GSE28829

We applied WGCNA to explore co-expression modules and hub genes [25]. The parameters chosen were R2 = 0.85, and the soft-threshold β = 14. The adjacency matrix was subsequently transformed into a topological overlap matrix (TOM). Modules were identified through hierarchical clustering with a minimum module size of 30. The eigengene and module membership (MM) were utilized to discern critical modules linked to ‘Early Atherosclerotic Plaque’ and ‘Advanced Atherosclerotic Plaque.’ ME represented the first principal component of the module and described its expression pattern, while MM indicated the relationship between genes and module eigengenes, reflecting the reliability of genes within modules. Functional enrichment analysis (Gene Ontology (GO) and Kyoto Encyclopedia of Genes and Genomes (KEGG) pathways) was conducted for genes within key modules using the “clusterProfile” R package.

### Enrichment-analysis

To better comprehend the molecular functionalities and pathway alterations within a specific subpopulation of smooth muscle cells, we conducted enrichment analysis based on DEGs (adjusted p-value < 0.05 and |logFC| > 0.75). This analysis primarily utilized the “clusterProfiler” R package [26]. The enrichment analysis included GO, KEGG (www.kegg.jp/kegg/kegg1.html), as well as gene set enrichment analysis (GSEA) [26–29].

### Hub gene selection and diagnostic model construction

To identify potential hub genes for diagnostic purposes, we focused on the genes that overlapped between the sets of differentially expressed genes from single-cell and transcriptome analyses, along with those identified as core module genes using WGCNA. We employed the LASSO and RF algorithms for feature selection and model construction.

The LASSO and RF methods were applied to screen and prioritize candidate diagnostic genes from this intersection. LASSO is a powerful method for feature selection in high-dimensional data, while RF is well-suited for handling complex, non-linear relationships within the data.

Moreover, to assess the diagnostic efficacy of the developed model, we computed the Area Under the Curve (AUC) using the ‘rROC’ R package. The AUC is a robust metric for assessing the model’s ability to discriminate between different groups and is commonly used to measure diagnostic accuracy.

### Construction of the rat carotid artery balloon injury model

Male Sprague-Dawley (SD) rats, with a weight range of 400–500 g, were sourced from Guangdong Laidi Biomedical Research Institute Co., Ltd. (Guangzhou, China) and were housed in appropriate animal facilities. All animal maintenance and handling procedures adhered to ARRIVE guidelines (https://arriveguidelines.org) and the protocols and guidelines established by the Institutional Animal Care and Use Committee of the First Affiliated Hospital of Sun Yat-sen University.

To establish the carotid balloon injury models, the rats were anesthetized through intraperitoneal injection of ketamine(100 mg/kg) and xylazine (10 mg/kg) mixed dilution. Subsequently, a midline neck incision was made to expose the left external carotid artery. Using a 2-F Fogarty arterial balloon catheter, the catheter was introduced into the left common carotid artery through the external carotid artery. The balloon was inflated and withdrawn three times to induce vascular injury. Following the removal of the catheter from the external carotid artery, the incision was sutured and closed.

After 14 days post-carotid artery injury, specimens were collected to represent the early-stage injury model. Specimens collected at 28 days post-injury were designated as the advanced-stage injury model. Samples from uninjured subjects were included as the healthy control group. The precise categorization of samples required microscopic observation of endothelial proliferation. This rat carotid artery balloon injury model serves as a valuable tool for studying vascular injuries and repair processes.

### Hematoxylin–eosin (H&E) staining

Frozen sections of rat carotid artery tissue were taken out from − 20℃ refrigerator and restored to room temperature, fixed with tissue fixative and then stained with hematoxylin stain for 5 min, differentiated with differentiation solution, returned to blue and then stained with eosin stain for 5 min, and sealed with neutral gum. The slices were examined microscopically and the images were collected and analyzed.

### Immunofluorescence staining

Immunofluorescence staining was employed to visualize the expression of *Igfbp4* and *Tagln* in frozen sections of rat carotid artery tissue. Primary antibodies used included a rabbit polyclonal antibody against *Igfbp4* (1:20 Cat No: 18500-1-AP, Proteintech) and a mouse monoclonal antibody against *Tagln* (1:500 Cat No: 60213-1-Ig, Proteintech). The staining procedure involved the following steps: Sections were permeabilized using Triton X-100 (0.1%) for 30 min. Subsequent to permeabilization, sections were washed with 1X PBST and blocked with 5% Fetal Bovine Serum for 1 h. Primary antibodies were then added to the blocking solution, and sections were incubated overnight at 4 °C on an orbital shaker. The following day, after three washes with PBS, sections were incubated in a blocking solution containing the secondary antibodies [CoraLite488-conjugated Goat Anti-Rabbit IgG(H + L) (1:200 Cat No: SA00013-2; Proteintech) and CoraLite594 – conjugated Goat Anti-Mouse IgG(H + L) (1:200 Cat No: SA00013-3; Proteintech)] for 1 h at room temperature. After three final washes with PBS, DAPI was used to stain the nuclei for 10 min. Finally, an anti-fluorescence attenuation sealant (MIKX, DB255, Shenzhen, China) was applied to a glass slide, and a cover glass slip was placed over the specimen. The stained sections were examined using an Olympus BX63 microscope.

### RT-qPCR

In this study, we conducted RT-qPCR to measure the expression levels of target genes in carotid arterial specimens. Total RNA was extracted from the carotid arterial specimens using the AG RNAex Pro Reagent (Cat No: AG21102; Accurate Biology). The RNA concentration was determined utilizing a Nanodrop 2000 spectrophotometer. RNA was reverse transcribed into cDNA using the Evo M-MLV Mix Kit (Cat No: AG11728; Accurate Biology), following the manufacturer’s instructions. qPCR was performed on a LightCycler 480 (Roche, Basel, Switzerland) instrument, utilizing the SYBR Green Premix Pro Taq HS qPCR Kit (Cat No: AG11701; Accurate Biology). The relative expression of target genes was estimated using the 2^(-ΔΔCt) method, with *Gapdh* as the reference control. The specific primer sequences used were as follows: *Igfbp4* (F: CTCCGCTCTGTGCTCTGTAG; R: CTAATCCCCCAGCACGAGTC) *Gapdh* (F: CAATCCTGGGCGGTACAACT; R: GATGGTGATGGGTTTCCCGT).

### Western blotting (WB)

In this study, we employed Western blotting to analyze protein expression in the tissue samples. Proteins were extracted from the tissue samples using a standard protein extraction protocol. SDS-PAGE: The extracted proteins were separated by sodium dodecyl sulfate-polyacrylamide gel electrophoresis (SDS-PAGE), allowing for the separation of proteins based on their molecular weight. After separation, the proteins were transferred from the gel to a polyvinylidene fluoride (PVDF) membrane. The PVDF membrane was blocked for 1 h at room temperature. It was incubated in Tris-buffered saline and Tween-20 (TBST) containing 5% nonfat milk powder. The PVDF membrane was then incubated with primary antibodies against the target proteins. In this case, the primary antibodies used were *Igfbp4* (1:1000 Cat No: 18500-1-AP, Proteintech) and *Alpha Tubulin* (1:3000 Cat No. 11224-1-AP, Proteintech). After primary antibody incubation, the membrane was washed three times with TBST to remove any unbound antibodies and other residues. The membrane was subsequently incubated for 1 h at room temperature with a secondary antibody. In this case, a horseradish peroxidase (HRP)-conjugated goat anti-rabbit secondary antibody (1:5000 Cat No: SA00001-2, Proteintech) was used. Immunoreactivity was detected employing an enhanced chemiluminescence (ECL) methodology, which allows the visualization of specific proteins based on the binding of HRP to its substrate. The Western blot images were captured using an automated digital gel image analysis system.

### Prediction of TFs and compounds

In this study, we aimed to predict TFs associated with *IGFBP4* and compounds interacting with *KLF15*. We employed the UCSC Genome Browser, accessible at https://genome.ucsc.edu/, in conjunction with the JASPAR database [30, 31]. This approach allowed us to predict potential TFs that may interact with *IGFBP4*. By analyzing the genomic data and binding sites available in the JASPAR database, we identified candidate TFs that may regulate *IGFBP4* expression.

For the prediction of compounds interacting with *KLF15*, we employed NetworkAnalyst 3.0 [32]. NetworkAnalyst 3.0 is a powerful visual analytics tool designed for network-based analysis. It enabled us to construct and explore networks representing interactions between KLF15 and various compounds.

### Statistical analysis

R software (version 4.2.2) and RStudio (version 2022.07.1 + 554) were used for data analysis and visualization. For comparing two groups, statistical comparisons were made using two-tailed Student’s t-test. The multiple comparisons were assessed by one-way analysis of variance (ANOVA) with Tukey’s test using GraphPad Prism 9.0 (California, USA). The p value < 0.05 was considered statistically.

## Results

### Quality control of single-cell data, dimensionality reduction, clustering, and cell type identification

**Fig. 1:**
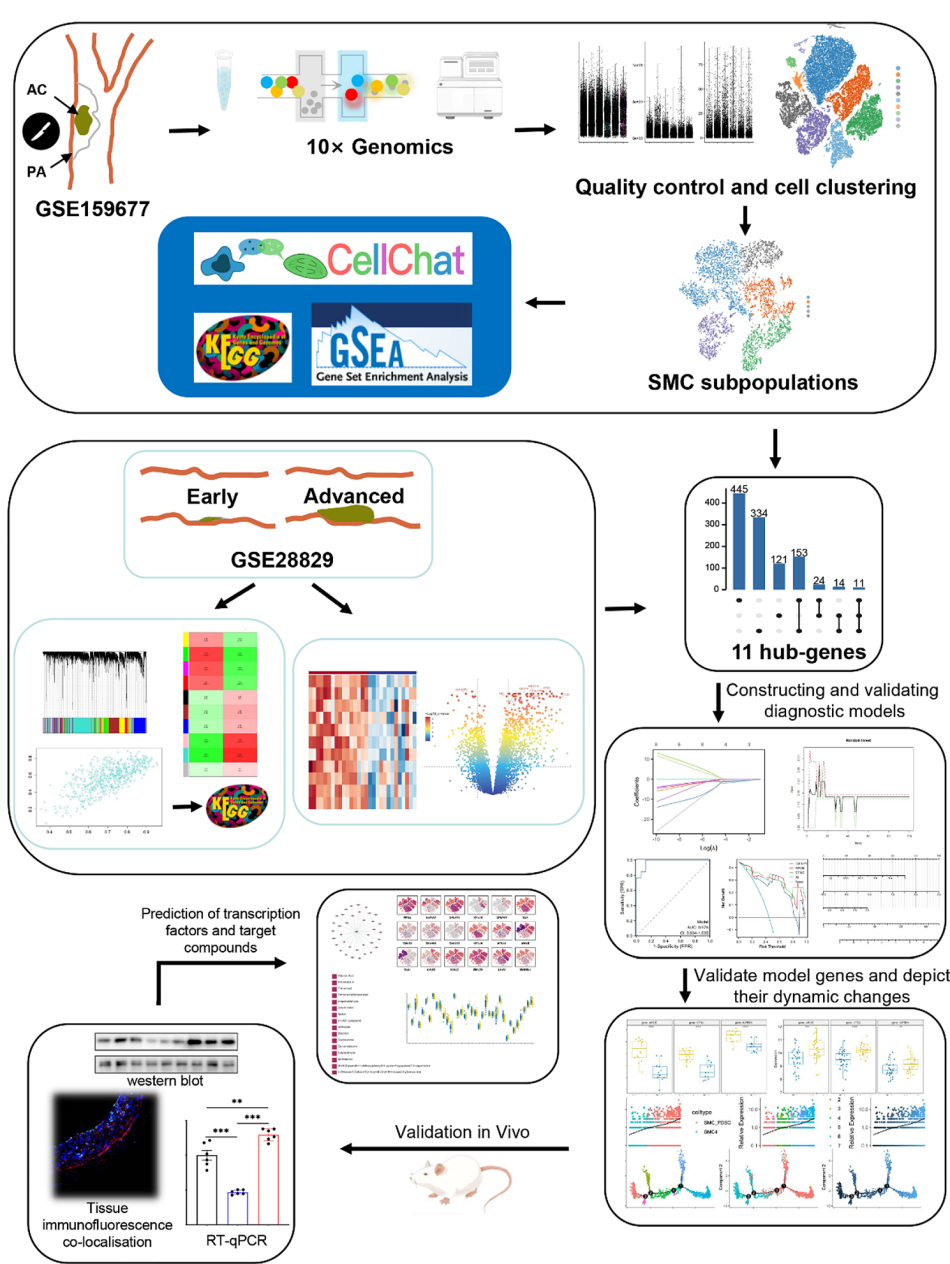
- Flowchart for the present research

**Fig. 2:**
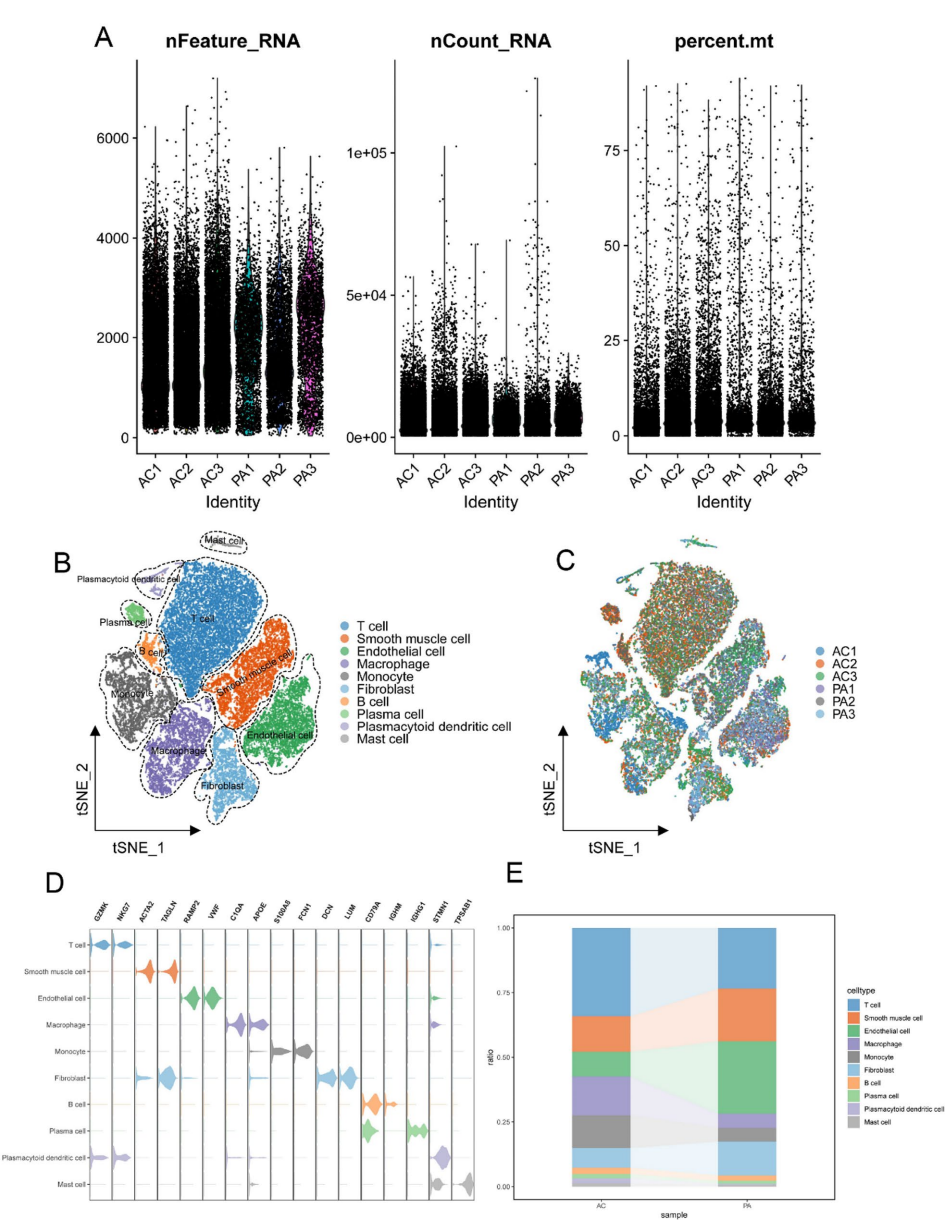
Data quality control and cell type identification. **A** Adjusting the mitochondrial ratio to ensure data quality. **B** Identifying 10 cell subpopulations, including T cell, smooth muscle cell, endothelial cell, macrophage, monocyte, fibroblast, B cell, plasma cell, plasmacytoid dendritic cell, mast cell. **C** Cell distribution within the samples. **D** Violin plot displaying the marker genes for each cell subgroup. **E** Displaying the proportions of each cell subgroup in two sample types

Figure 1 provides an overview of the workflow for this study (Fig. 1). In the case of the single-cell dataset GSE159677, the first step involved quality control measures. We excluded specific cells and managed the proportion of mitochondrial genes to ensure the quality of the cell samples used in this study (Fig. 2A). Following quality control, a total of 40,953 cells were retained. Through cell clustering and annotation, we identified ten distinct cellular subgroups, including T cells, smooth muscle cells, endothelial cells, macrophages, monocytes, fibroblasts, B cells, plasma cells, plasmacytoid dendritic cells, and mast cells (Fig. 2B-C). Figure 2D illustrates the important marker genes associated with each cell type (Fig. 2D). Figure 2E demonstrates the proportional distribution of each cell type in two different samples (Fig. 2E).

### Identification of smooth muscle cell subgroups, analysis of cell-to-cell interactions within these subgroups and enrichment analysis

**Fig. 3:**
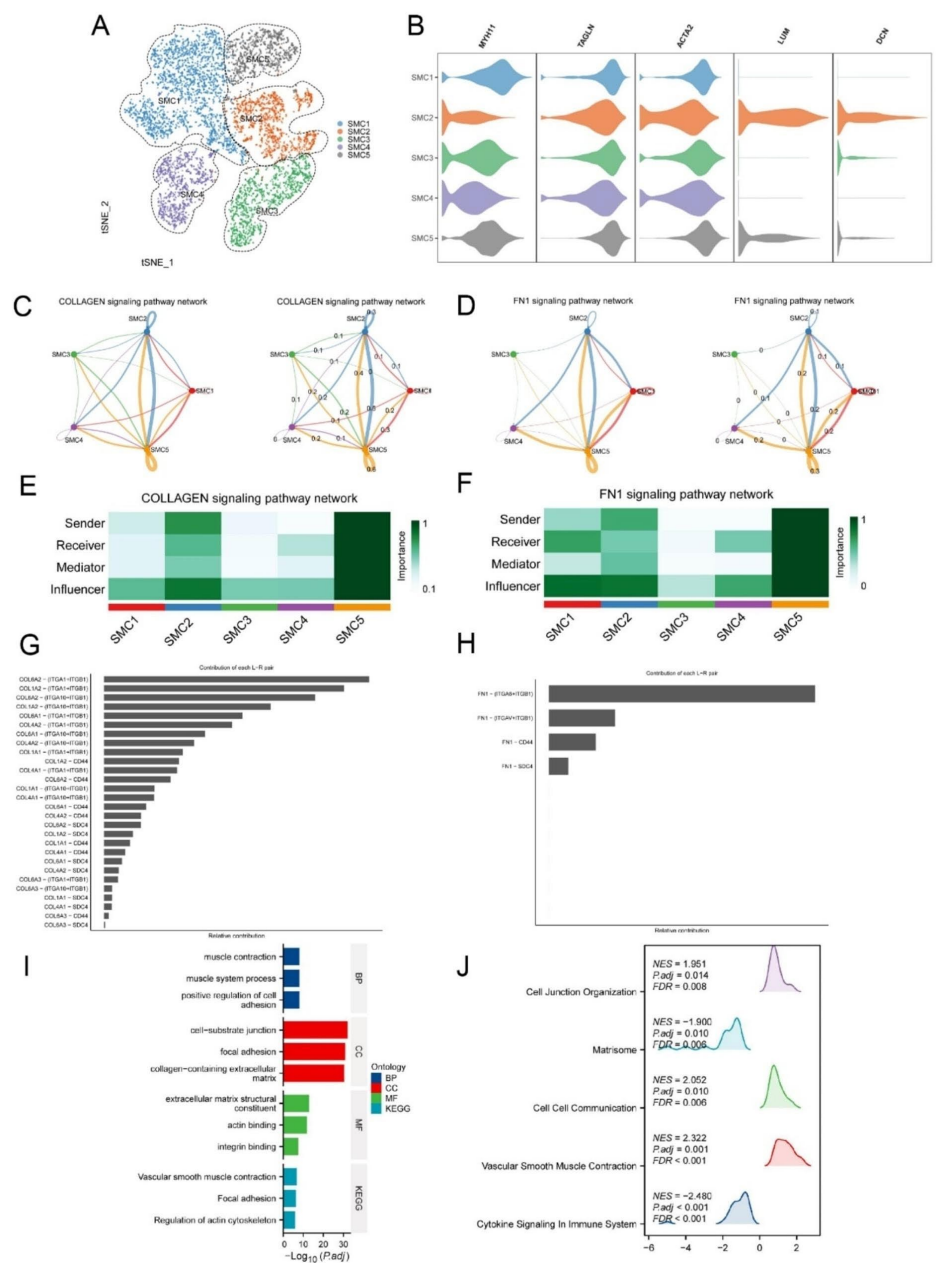
Cell communication and enrichment analysis among smooth muscle cell subtypes. **A** Identifying 5 smooth muscle cell subpopulations, including SMC1, SMC2, SMC3, SMC4, SMC5. **B** Violin plot showing the five smooth muscle cell marker genes. **C-D** The network diagram illustrating the interaction network between smooth muscle cell subtypes in the COLLAGEN and FN1 signaling pathway. **E-F** The cell communication heatmap displaying the role of SMC2 and SMC5 in the COLLAGEN and FN1 signaling pathway. **G-H** Relative contribution of each ligand–receptor pair as it affects the overall communication network of the COLLAGEN and FN1 signaling pathway. **I** Bar chart displaying the results of GO and KEGG enrichment analysis for differentially expressed genes between SMC2 and SMC5. **J** Mountain plots presenting the results of GSEA analysis for differentially expressed genes between SMC2 and SMC5

The biological behavior of smooth muscle cells within atherosclerotic plaques is closely associated with the stability of the plaques. Therefore, we extracted 6261 smooth muscle cells from the dataset and performed a re-clustering to categorize them. Additionally, we conducted cellular communication analysis among subgroups of smooth muscle cells. SMC2 cluster displays significant heterogeneity among smooth muscle cells, characterized by high expression of fibroblast typical markers LUM and DCN, we refer to this as fibroblast-like smooth muscle cells (Fig. 3A and B). To identify the initiating genetic factors in the transition toward fibroblast-like smooth muscle cells, we conducted an analysis of cell-cell interactions within the subgroups. We discovered that SMC2 and SMC5 may interact in the COLLAGEN and FN1 pathways (pro > 0.2, p-value < 0.05). Figure 3C shows the number and interaction weight of COLLAGEN signaling pathway (Fig. 3C). Figure 3D shows the number and interaction weight of FN1 signaling pathway (Fig. 3D). Figure 3E and F shows the role of SMC2 and SMC5 in the COLLAGEN and FN1 signaling pathway (Fig. 3E and F). In the COLLAGEN signaling pathway, the most significant receptor-ligand pair is COL6A2-(ITGA1 + ITGB1) (Fig. 3G); while in the FN1 signaling pathway, the most prominent contribution comes from FN1-(ITGA8 + ITGB1) (Fig. 3H). To gain a better understanding of the potential mechanisms and pathways where interactions may occur between SMC2 and SMC5, we conducted enrichment analysis (GO, KEGG, and GSEA) on differentially expressed genes in both groups of SMCs. The GO enrichment analysis results indicate that biological processes (BP) are mainly associated with positive regulation of cell adhesion, muscle contraction and muscle system process. Cellular components (CC) are primarily related to cell-substrate junction, focal adhesion and collagen-containing extracellular matrix. Molecular functions (MF) are mainly associated with extracellular matrix structural constituent, actin binding and integrin binding. KEGG pathway enrichment reveals significant associations with vascular smooth muscle contraction, focal adhesion, and regulation of actin cytoskeleton pathways (Fig. 3I). GSEA enrichment analysis reveals significant associations with cell junction organization, matrisome, cell-cell communication, vascular smooth muscle contraction, cytokine signaling in immune system. This comprehensive analysis offers valuable insights into the potential mechanisms underpinning the interactions between SMC2 and SMC5, shedding light on their roles in the context of atherosclerotic plaques.

### The comprehensive analysis of GSE28829

To comprehensively elucidate the mechanisms underlying plaque stability from multiple perspectives, we conducted both WGCNA and differential expression analysis at the transcriptome level using the GSE28829 dataset. This approach, in conjunction with our single-cell analysis, allowed us to pinpoint the key driver genes more accurately.

**Fig. 4:**
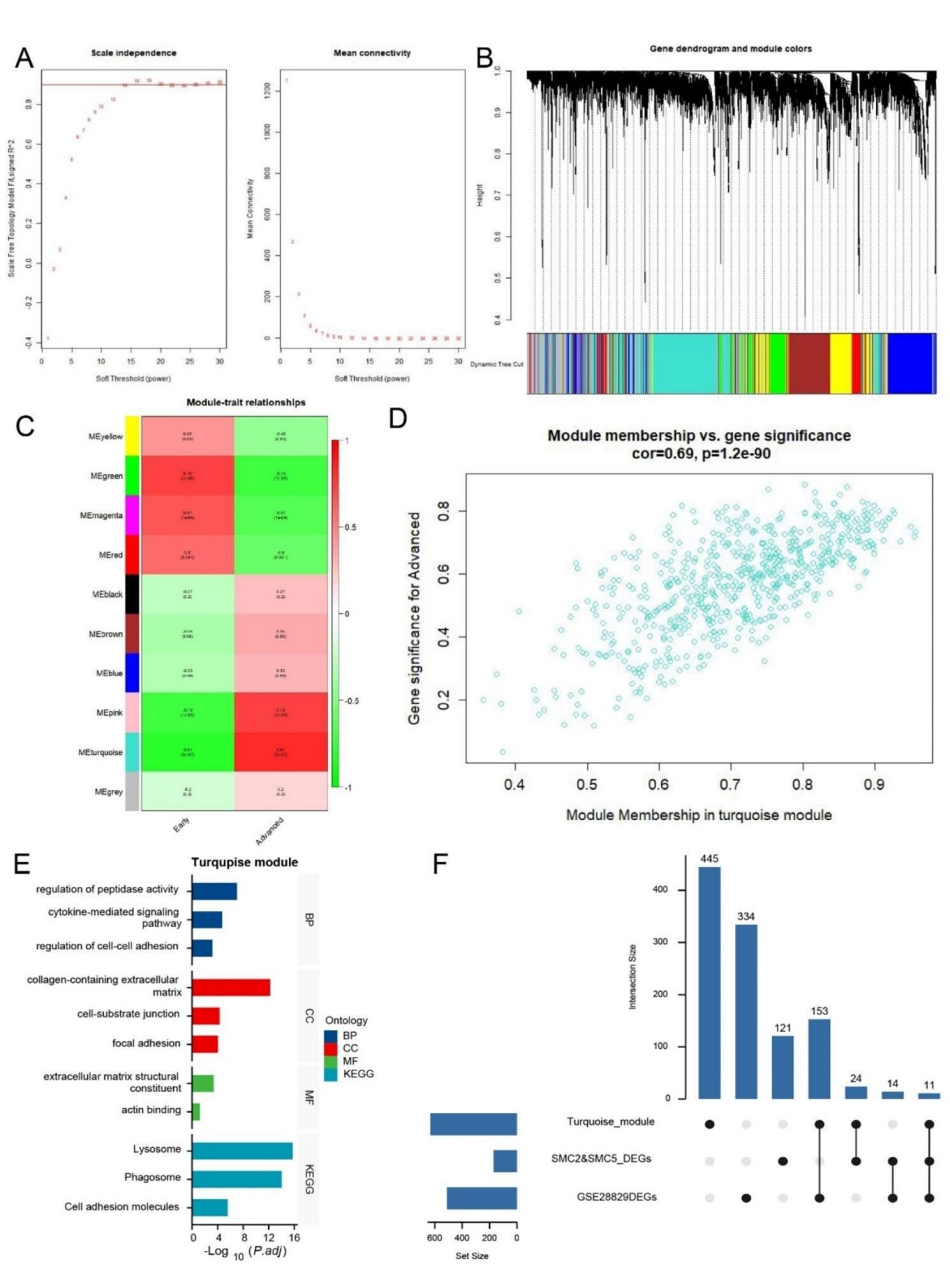
WGCNA analysis of GSE28829 and functional enrichment analysis of key module. **A** Selection of Soft Threshold 14. **B** Genes with strong correlations grouping into the same module, forming modules of different colors. The turquoise module representing a relatively large proportion. **C** Correlation analysis between modules and advanced atherosclerotic plaques. The turquoise module showing the highest correlation (cor = 0.81, *p* = 0.000003). **D** Scatter plot analysis of the turquoise module. **E** Presentation of the GO and KEGG enrichment results for the turquoise module. **F** UpSet plot showing the intersection of differentially expressed genes identified in single-cell analysis, genes in the turquoise module, and differentially expressed genes in transcriptome analysis. The intersection of these analyses including 11 genes

After a rigorous data preprocessing step, we identified and removed an outlier sample. We then calculated the mean expression levels of the top 5000 genes. The power parameter was carefully optimized using the “pickSoftThreshold” function from the “WGCNA” package. A power value of β = 14 (corresponding to a scale-free R^2 of 0.9) was chosen as the soft threshold, facilitating the construction of a scale-free network (Fig. 4A).

By employing a combination of average hierarchical clustering and dynamic tree clipping, we identified a total of 10 modules (Fig. 4B). To uncover the modules with clinical significance, we examined their associations with disease states. Among these 10 modules, the turquoise module exhibited the strongest correlation with advanced plaque (correlation coefficient = 0.81 and p-value = 3e-07) (Fig. 4C). As a result, we focused our further analysis on the turquoise module, which includes 633 key genes (Fig. 4D).

In-depth analysis of the genes within the turquoise module involved GO and KEGG enrichment analysis. The findings revealed that in terms of BP, this module is primarily associated with the regulation of peptidase activity, the cytokine-mediated signaling pathway, and the regulation of cell-cell adhesion. Regarding CC, the key associations are with cell-substrate junctions, focal adhesions, and collagen-containing extracellular matrices. MF are predominantly linked to extracellular matrix structural constituents and actin binding. KEGG enrichment analysis uncovered significant links with the lysosome, phagosome, and cell adhesion molecules pathways (Fig. 4E).

**Fig. 5:**
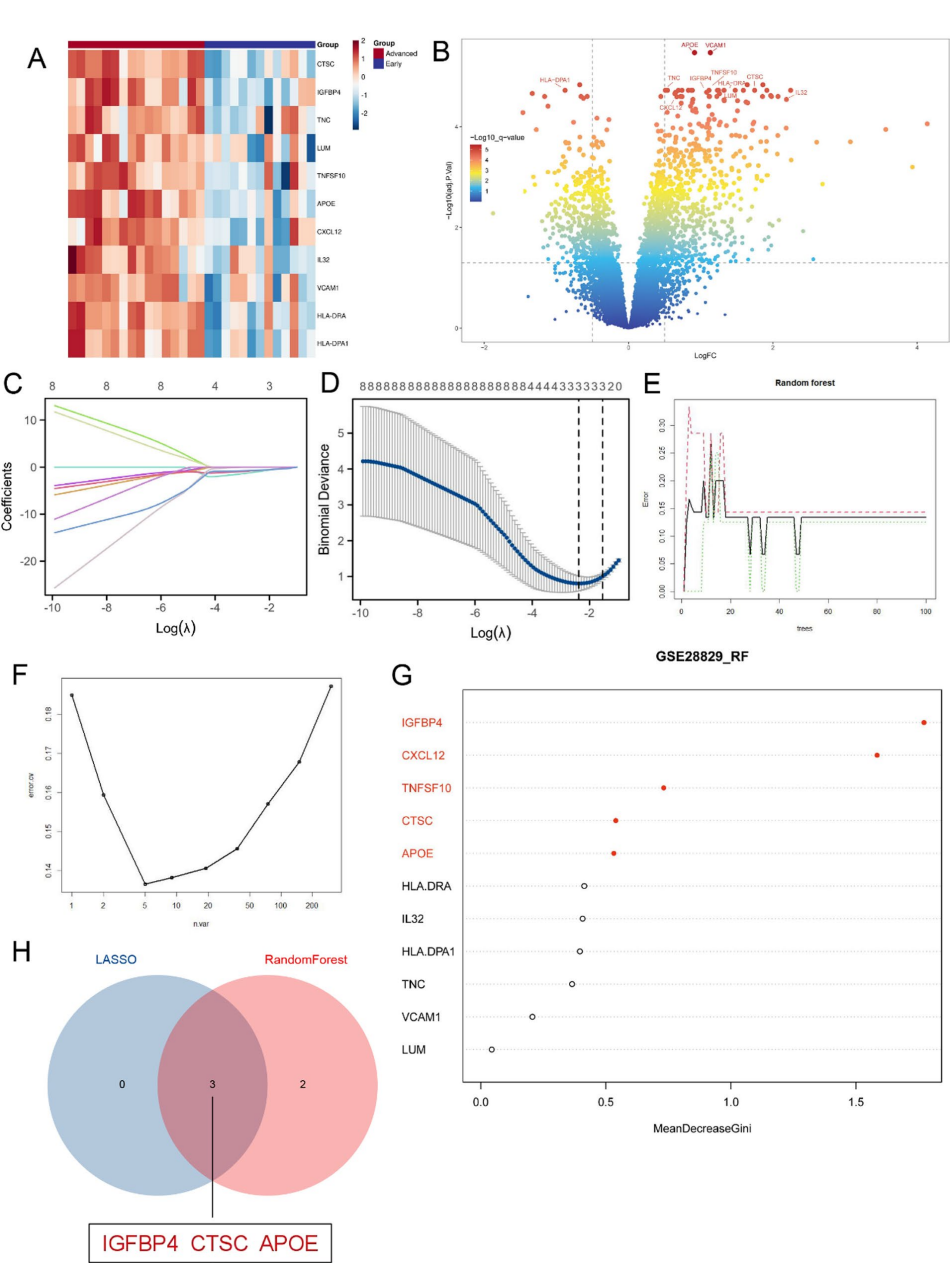
Transcriptome differential expression analysis of GSE28829 and gene selection using machine learning algorithms. **A** Heatmap plot showing the 11 intersecting genes in the transcriptome differential expression data. **B** The volcano plot displaying the differentially expressed genes from the transcriptome differential expression analysis, with the 11 intersecting genes labeled. **C-D** Feature selecting through LASSO algorithm. **E** The influence of the number of decision trees on the error rate. The X-axis represents the number of decision trees, while the Y-axis represents the error rate. **F** Selecting an appropriate number of gene variables based on error rate. The x-axis represents the number of gene variables, and the y-axis represents the error rate. **G** The results of the Gini coefficient method in the RF classifier. The top 5 genes marking in red. **H** Venn diagram showing the shared genes between the LASSO and RF algorithms

Furthermore, we conducted differential analysis once more on GSE28829, identifying 512 significantly differentially expressed genes (|logFC| > 0.75, adjusted p-value < 0.05). These genes were then intersected with those identified in the single-cell analysis and the hub genes from the WGCNA, ultimately yielding 11 hub genes (Fig. 4F). The heatmap displays these 11 intersecting genes (Fig. 5A), and the volcano plot illustrates the significantly differentially expressed genes resulting from the differential analysis (Fig. 5B).

### Further screening of hub genes using RF and LASSO algorithms

To identify potential biomarkers for distinguishing advanced plaques, we employed two distinct machine learning algorithms. First, utilizing the LASSO regression algorithm, we refined the list of hub genes from 11 to 3 (Fig. 5C and D). Subsequently, we implemented the RF algorithm, which demonstrated the lowest error rate when the gene set was further narrowed down to 5, based on their importance (Fig. 5E and G).

The hub genes that intersected in both the LASSO and RF algorithms, namely *IGFBP4*, *CTSC*, and *APOE*, were selected for further in-depth investigation. These genes hold the potential to serve as critical markers for the identification of advanced plaques.

### Construction and validation of a diagnostic model for advanced plaque based on hub genes

**Fig. 6:**
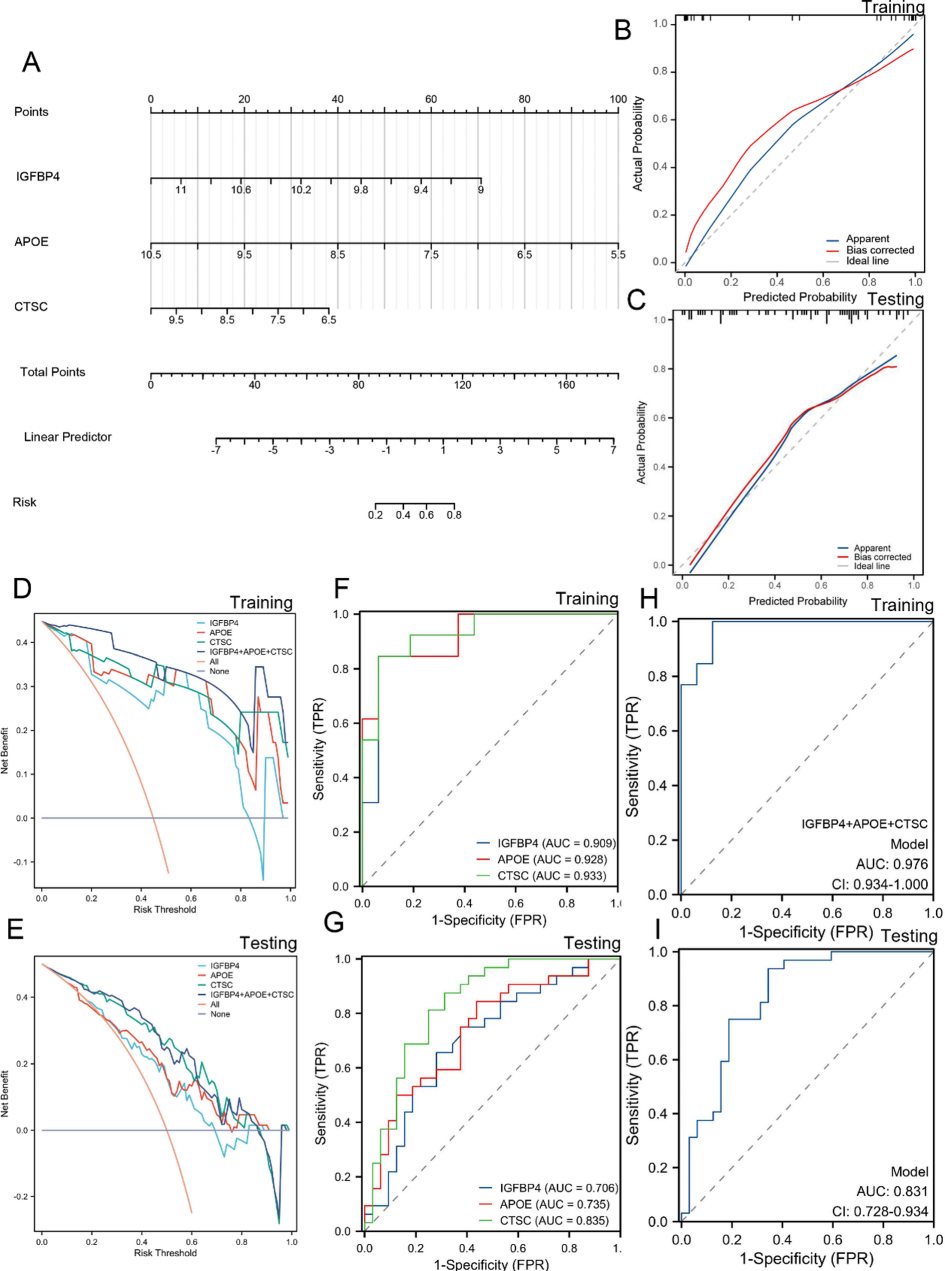
Building and validating nomograms. **A** Construction of the nomogram model. The calibration curve of the nomogram in **B** training and **C** testing sets. DCA curve for assessing individual genes and diagnostic model in **D** training and **E** testing sets. ROC curve for individual genes in **F** training and **G** testing sets. ROC curve for model in **H** training and **I** testing sets

Firstly, we utilized hub genes (*IGFBP4, CTSC, APOE*) to construct a diagnostic model, specifically a Nomogram model, for advanced plaque (Fig. 6A). The calibration curve was employed to evaluate the predictive performance of the Nomogram model in both the training (GSE28829) and testing (GSE43292) datasets. These two calibration curves revealed a small discrepancy between the actual risk and predicted risk for advanced plaque, indicating that the Nomogram model possesses high accuracy (Fig. 6B and C). Upon conducting Decision curve analysis (DCA), it became evident that the curve for the combination “*IGFBP4* + *APOE* + *CTSC*” surpassed the curves representing “No intervention for all”, “Intervention for all”, and all individual genes. This observation indicates that patients may derive significant benefits from the nomogram model within a high-risk threshold ranging from 0 to 1. Additionally, the clinical advantage offered by the nomogram model was notably superior when compared to the curve generated by individual genes (Fig. 6D and E). Subsequently, we employed ROC curves to evaluate the diagnostic performance of individual genes and the model. In the training set, the AUC values for *IGFBP4*, *APOE*, and *CTSC* were 0.909, 0.928, and 0.933, respectively, while for the model, it was 0.976. In the testing set, the AUC values for *IGFBP4*, *APOE*, and *CTSC* were 0.706, 0.735, and 0.835, respectively, and for the model, it was 0.831 (Fig. 6F - I). These results demonstrate that both individual genes and the diagnostic model play a significant role in advanced plaques.

### Validation of model genes and In-depth exploration of *IGFBP4*

**Fig. 7:**
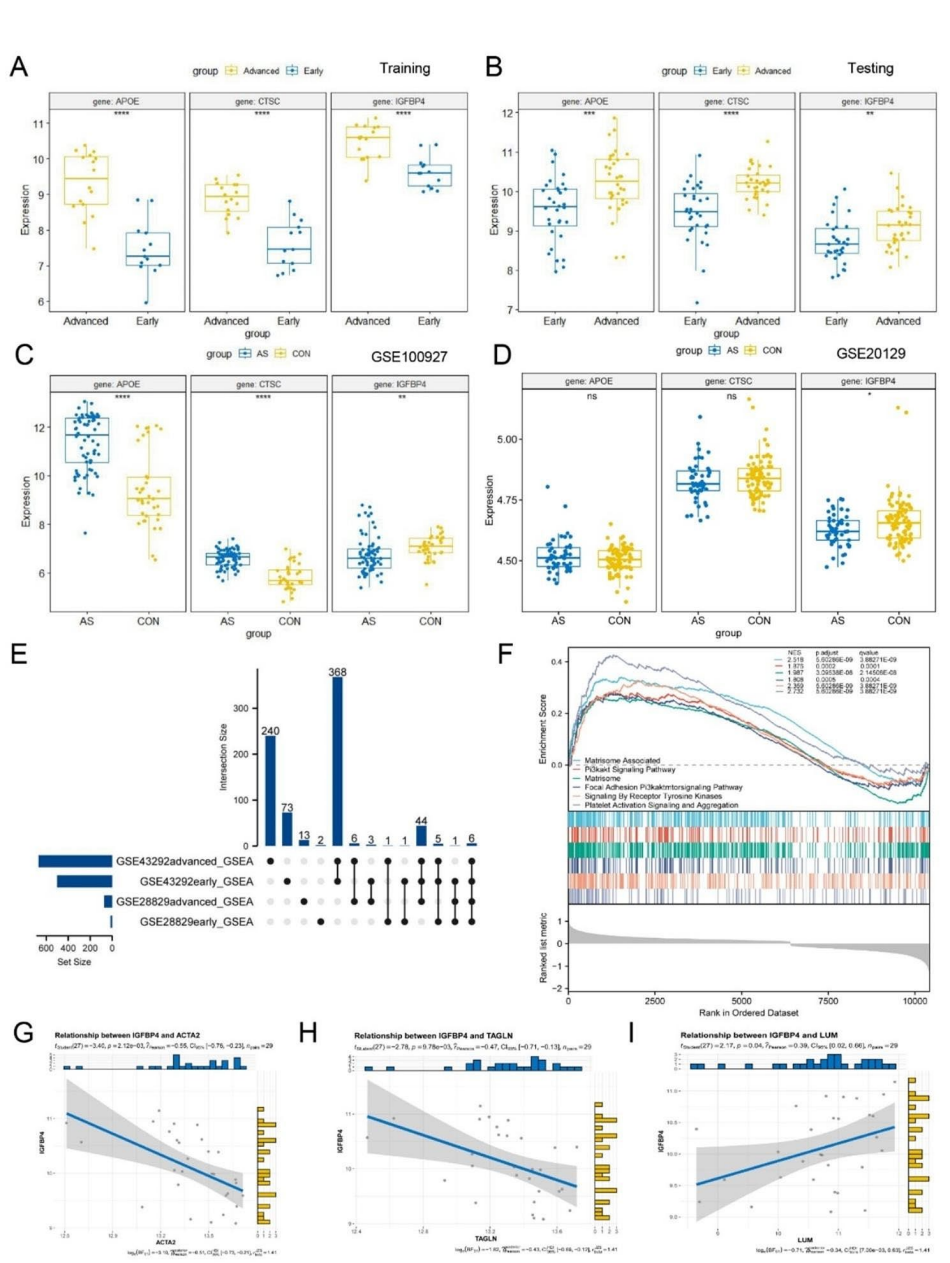
Model genes expression validation; GSEA enrichment analysis, and correlation analysis based on *IGFBP4*. The relative expression levels of *IGFBP4*, *CTSC* and *APOE* in **A** training and **B** testing sets. The relative expression levels of *IGFBP4*, *CTSC* and *APOE* in **C** GSE100927 and **D** GSE20129. **E** UpSet plot showing shared pathways in the GSEA enrichment analysis of early and advanced plaques from GES28829 and GSE43292, which based on the expression of *IGFBP4*. **F** The shared GSEA enrichment pathways based on the expression of *IGFBP4*. The scatterplot displaying the correlation between *IGFBP4* and **G***ACTA2*, **H***LUM*, **I***TAGLN*. (*p < 0.05; **p < 0.01; ***p < 0.001****; *p* < 0.0001; ns, no statistical significance)

Following the comprehensive analysis conducted earlier, three genes—*IGFBP4*, *CTSC*, and *APOE*—were identified. To validate these genes, we examined their expression in both the training dataset (GSE28829) and an external testing dataset (GSE43292). The results indicated significant statistical differences in gene expression between early and advanced plaques in both the training and testing sets (Fig. 7A-B).

Additionally, we validated these genes in samples from both healthy and atherosclerotic individuals. Intriguingly, only *IGFBP4* displayed significant differences in expression, both in blood and surgically excised tissue samples, with high expression in healthy tissues (Fig. 7C-D). These findings suggest that *IGFBP4* may have a protective role. It implies that *IGFBP4* could not only protect blood vessels from atherosclerosis but also potentially promote plaque stability, especially in the advanced stages of atherosclerosis.

Based on the training and testing datasets, the GSEA of *IGFBP4* gene expression levels indicated its potential involvement in signaling pathways such as PI3K-AKT and Matrisome-associated pathways, among others (Fig. 7E-F).

Furthermore, we conducted a correlation analysis between *IGFBP4* and marker genes for smooth muscle cells (*ACTA2*, *TAGLN*) and fibroblasts (*LUM*). The analysis revealed a negative correlation between *IGFBP4* expression and smooth muscle cell marker genes, while demonstrating a positive correlation with fibroblast marker genes (Fig. 7G-I). In summary, *IGFBP4* is of paramount importance and may play a role in driving smooth muscle cells toward a transition to fibroblast-like smooth muscle cells.

### Analysis of gene dynamics in pseudotime

**Fig. 8:**
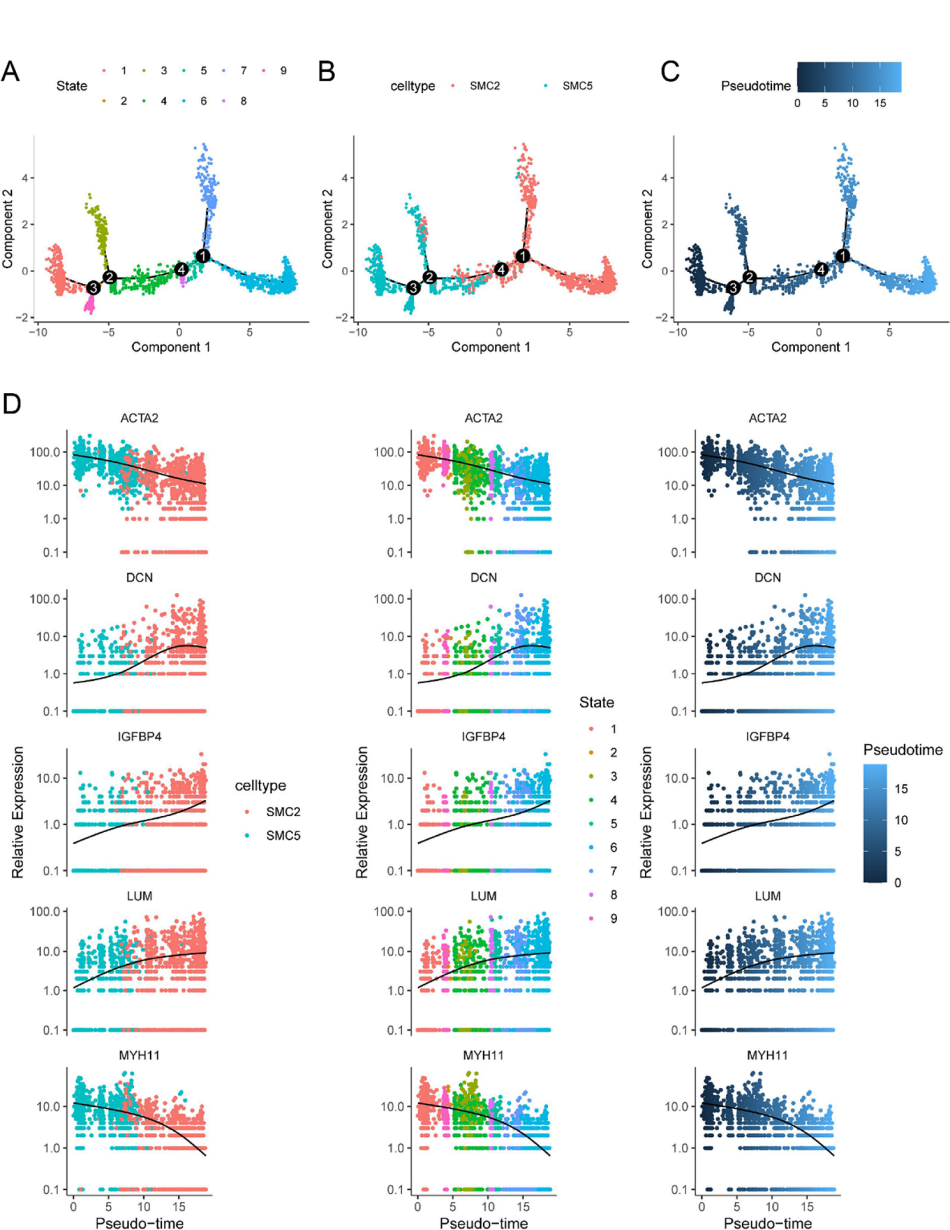
Pseudotime analysis between SMC2 and SMC5 clusters. **A** Nine stages of SMC differentiation. **B** The SMC2 and SMC5 cells using for pseudo-time analysis. **C** The temporal changing in cell differentiation. **D** The dynamic expression of *IGFBP4* and marker genes along pseudotime

To gain deeper insights into the dynamic changes of *IGFBP4* and cell marker genes in the SMC2 and SMC5 subgroups, we performed pseudotime analysis. Our analysis revealed that, as time progressed, the SMC5 subgroup showed a differentiation trend towards the SMC2 subgroup (Fig. 8A-C).

During this differentiation process, the expression of *IGFBP4* exhibited a gradual increase over time. This increase in *IGFBP4* expression was accompanied by an elevation in the expression of fibroblast marker genes such as *LUM* and *DCN*, while there was a concurrent decrease in the expression of smooth muscle cell marker genes including *ACTA2* and *MYH11* (Fig. 8D).

These findings from the dynamic gene analysis within the SMC subgroups are consistent with our previous correlation analysis in the transcriptomic data. They further emphasize the pivotal role of *IGFBP4* in driving the transition of smooth muscle cells towards a fibroblast-like phenotype.

### Validation of *IGFBP4* in animal models

Our integrated analysis of single-cell and transcriptomic data in human carotid artery samples indicated a significant role for *IGFBP4* in smooth muscle cells. To strengthen the credibility of these findings, we conducted in animal experiments using a rat carotid artery balloon injury model. We classified the model into three groups: normal, early mild hyperplasia, and advanced severe hyperplasia based on post-injury time and microscopic intimal hyperplasia thickness.

**Fig. 9:**
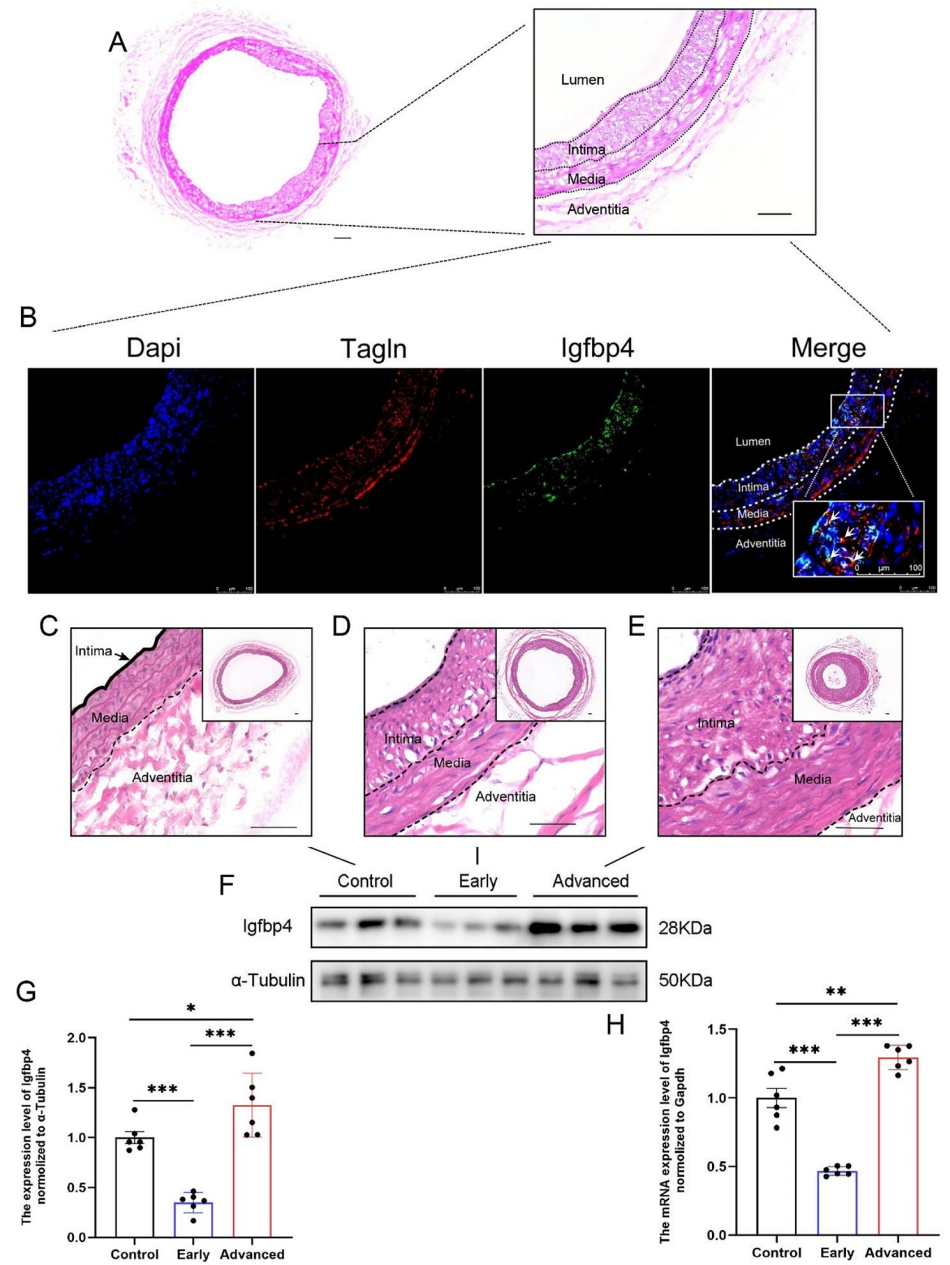
Experimental validation of *Igfbp4* expression. **A** Hematoxylin & eosin (HE) staining showing the macroscopic morphology and local magnification of vascular anatomical layers in rat carotid artery after balloon injury. (scale bar = 50 μm) **B** Localization of *Igfbp4* expression in injury artery. HE Staining displaying the macroscopic and localized vascular landscapes of **C** normal artery, **D** early injured artery, and **E** advanced injured artery. (scale bar = 50 μm) **F-G** WB validating the protein expression levels of *Igfbp4* in normal arteries, early injured arteries, and advanced injured arteries, using *α-Tubulin* as the housekeeping protein for normalization. **H** RT-qPCR experiment validating the mRNA expression levels of *Igfbp4* in normal arteries, early injured arteries, and advanced injured arteries, using *Gapdh* as the housekeeping gene for normalization. (*p < 0.05; **p < 0.01; ***p < 0.001)

First, we performed immunofluorescence co-localization of *Igfbp4* and observed its presence in the hyperplastic intima, co-localizing with the smooth muscle cell-specific marker, *Tagln* (Fig. 9A-B).

Additionally, we extracted tissue RNA and protein from these three groups (Fig. 9C-E) and assessed *Igfbp4* expression. The results demonstrated that *Igfbp4* expression was lower in the early mild intimal hyperplasia group compared to the normal group and higher in the advanced severe hyperplasia group compared to the normal group (Fig. 9F-H). These differences were statistically significant. These experimental results align with our bioinformatics analysis and provide further support for the role of *IGFBP4* in both normal and atherosclerotic populations.

### Prediction of TFs and TF-binding compounds

**Fig. 10:**
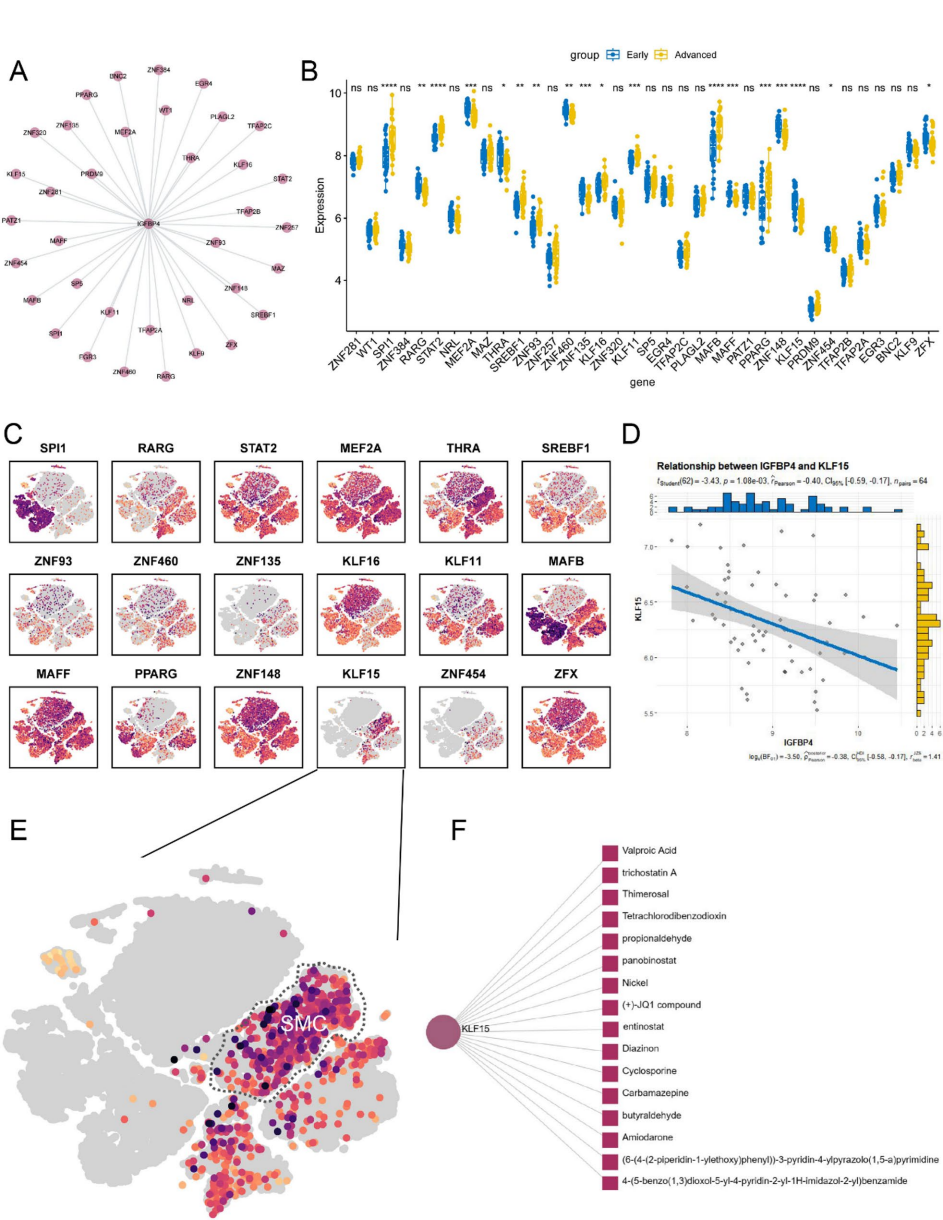
The prediction of TFs and targeted compound. **A** Network diagram of TFs binding with *IGFBP4*. **B** Box plot depicting the differences in TFs expression between early and advanced plaques. **C** The t-SNE plot displaying the expression level and localization of TFs in cell clusters. **D** The scatter plot showing the correlation between *KLF15* and *IGFBP4*. **E** The enlarged t-SNE plot for *KLF15*. **F** The network diagram of compounds acting on *KLF15*. (*p < 0.05; **p < 0.01; ***p < 0.001)

Given the tissue and cell-specific expression patterns of TFs, we conducted predictions of potential TFs that might bind to *IGFBP4*. This analysis revealed several candidates, including *BNC2, EGR3, EGR4, KLF11, KLF15, KLF16, KLF9, MAFB, MAFF, MAZ, MEF2A, NRL, PATZ1, PLAGL2, PPARG, PRDM9, RARG, SP5, SPI1, SREBF1, STAT2, TFAP2A, TFAP2B, TFAP2C, THRA, WT1, ZFX, ZNF135, ZNF148, ZNF257, ZNF281, ZNF320, ZNF384, ZNF454, ZNF460, ZNF93* (Fig. 10A).

To identify TFs with differential expression and cell subtype specificity within vascular smooth muscle cells, we compared datasets containing samples from early and advanced plaques (GSE43292). Our analysis pinpointed *KLF15* as the sole candidate meeting the criteria (Fig. 10B, C and E) and exhibiting a negative correlation with *IGFBP4* (Fig. 10D).

Furthermore, we utilized the NetworkAnalyst database to predict compounds that could potentially modulate *KLF15.* The results unveiled that several compounds had the capacity to influence *KLF15* mRNA expression significantly. These compounds include 4-(5-benzo (1,3) dioxol-5-yl-4-pyridin-2-yl-1 H-imidazol-2-yl) benzamide, (6-(4-(2-piperidin-1-ylethoxy) phenyl))-3-pyridin-4-ylpyrazolo(1,5-a) pyrimidine, Amiodarone, butyraldehyde, Carbamazepine, Cyclosporine, Diazinon, entinostat, (+)-JQ1 compound, Nickel, panobinostat, propionaldehyde, Tetrachlorodibenzodioxin, Thimerosal, and trichostatin A (Fig. 10F).

These findings provide valuable insights into potential TFs associated with *IGFBP4* and compounds that may significantly impact *KLF15* mRNA expression.

## Discussion

The destabilization and rupture of atherosclerotic plaques stand out as primary contributors to cardiovascular ischemic events. Addressing these challenges by identifying, intervening, and reversing early-stage plaques, alongside stabilizing advanced unstable plaques, has become pivotal in contemporary atherosclerosis treatment. A significant portion of atherosclerotic plaque lesions comprises heterogeneous cells originating from smooth muscle [33]. The progression and stability of atherosclerotic plaques are profoundly shaped by smooth muscle cells, which undergo phenotype transformations in response to relevant pathological pressures [34, 35]. Consequently, modifications in smooth muscle cell biology play a pivotal role in the initiation and progression of atherosclerosis. Notably, fibroblast-like smooth muscle cells, characterized by their synthetic phenotype and production of extracellular matrix, emerge as key contributors to the formation of fibrous caps, thereby promoting plaque stability [7]. Consequently, fostering the transformation of smooth muscle cells into fibroblast-like cells proves instrumental in fortifying plaque stability. This strategic shift in cell phenotype offers a promising avenue for therapeutic interventions aimed at enhancing the resilience and stability of atherosclerotic plaques.

The emergence of single-cell analysis techniques has empowered us with the capability to gain a nuanced understanding of the intricacies within plaques [36]. Our study embarked on a direct analysis of single-cell data sourced from human carotid artery samples, distinguishing itself from previous endeavors conducted at the animal level [37]. In the course of re-clustering the smooth muscle cell cluster, our scrutiny unearthed a remarkably heterogeneous subset of smooth muscle cells, coined SMC2. This subset displayed heightened expression levels of *LUM* and *DCN*, recognized markers for fibroblasts, indicative of a fibroblast-like smooth muscle cell subgroup, warranting our attention.

To unravel the determinants steering the transition of smooth muscle cells towards the SMC2 subgroup, we executed intercellular communication analysis among the smooth muscle cell subgroups. Our findings spotlighted that, among pathways with a probability of intercellular communication surpassing 0.2, only the COLLAGEN and FN1 signaling pathways exhibited significant interactions between the SMC2 and SMC5 subgroups. Subsequent differential expression analysis and functional enrichment analysis between these subgroups brought to light substantial associations with processes related to the extracellular matrix, cell adhesion, and vascular smooth muscle contraction, among other factors.

To bolster the credibility of our single-cell level enrichment analysis, a parallel WGCNA at the transcriptome level was executed using the GSE28829 dataset. This analytical effort aimed to pinpoint the turquoise module, most pertinent to advanced plaques. The enrichment analysis of this module underscored close associations with the extracellular matrix, cell adhesion, and additional factors. In tandem, a secondary analysis of GSE28829 utilizing differential expression analysis was performed. By intersecting the differentially expressed genes garnered from these three analytical approaches, we identified 11 candidate genes poised to potentially play a pivotal role in fortifying plaque stability.

In the current landscape, the widespread adoption of machine learning algorithms has become pivotal in selecting genes for the development of disease diagnosis and prognosis models [38]. Taking this into account, we advanced our investigation by subjecting the 11 identified candidate genes to screening using both the LASSO and RF algorithms. This comprehensive approach culminated in the construction of a diagnostic model meticulously designed to assess plaque conditions. External validation procedures substantiated the superior diagnostic performance of both individual genes and the composite model, prominently featuring *IGFBP4/APOE/CTSC*. This outcome profoundly underscores the indispensable role played by these three genes in the intricate landscape of atherosclerosis.

To further corroborate our findings, we meticulously validated the expression levels of the model genes within our dataset. Intriguingly, we noted higher expressions of these genes in advanced plaques compared to early plaques, irrespective of their stability. Notably, in both the normal and atherosclerosis groups (comprising both plaque tissue and blood samples), a distinctive pattern emerged. Specifically, *IGFBP4* exhibited elevated expression levels in the normal group, in stark contrast to *APOE* and *CTSC*. This observation indicates a unique role for *IGFBP4* in plaque dynamics.

Moreover, our correlation and pseudotemporal gene dynamics analyses yielded compelling insights. Changes in *IGFBP4* expression were negatively correlated with smooth muscle cell-associated marker genes while positively correlated with fibroblast-associated marker genes. This intricate interplay suggests that the heightened expression of *IGFBP4* in advanced lesions may act as a driving force in the conversion of smooth muscle cells into fibroblast-like smooth muscle cells, underscoring its crucial role in the complex trajectory of plaque progression.

Consequently, we focused our study on *IGFBP4*. Given the high conservation of the human *IGFBP4* gene with splice sites in the rat *Igfbp4* gene [39], we conducted an investigation of *Igfbp4* in a rat carotid artery balloon injury model. The results demonstrated its co-localization with smooth muscle cells within proliferative intimal tissue. Furthermore, the expression trend of *IGFBP4* in rat arterial tissue, ranging from normal to early and advanced intimal proliferation, was consistent with the results of our bioinformatics analysis.

*IGFBP4*, belonging to the insulin-like growth factor-binding protein family, primarily functions by inhibiting the action of IGF through the prevention of IGF binding to its receptor [40, 41]. Although studies focusing on *IGFBP4* in the field of atherosclerosis research have been relatively scarce, there have been reports of *IGFBP4* levels affecting various biological behaviors, particularly in cancer-related diseases. For instance, changes in *IGFBP4* levels have been linked to tumor cell proliferation in lung cancer and have shown associations with prognosis [42–45]. Notably, *IGFBP4* exhibits widespread expression in both blood and tissues, and interventions targeting *IGFBP4* may lead to unforeseen side effects.

TFs play a pivotal role in finely regulating transcriptional processes within specific cell types, orchestrating them spatially and temporally to induce or maintain specific cellular fates. This process profoundly influences the phenotypic characteristics of organisms [46]. Our predictions regarding TFs for *IGFBP4* revealed that *KLF15* has specificity for subtypes of smooth muscle cells and possesses the potential to bind to *IGFBP4*. Among the Krüppel-like family of TFs, which are central to the regulation of the cardiovascular system [47], *KLF15* has been identified as a key regulator of vascular smooth muscle cells [48, 49]. Consequently, the regulation of VSMCs by *KLF15/IGFBP4* may hold significant importance in promoting plaque stability. Furthermore, we have identified compounds predicted to interact with *KLF15*, which may serve as candidates for drugs aimed at enhancing plaque stability.

In summary, we propose the following hypothetical scenario: In the vascular system of healthy individuals, relatively high levels of *IGFBP4* help maintain the normal physiological state of vascular smooth muscle cells. However, in the early stages of vascular pathology, *KLF15*, acting as a regulator of smooth muscle cells, begins to upregulate in response to pathological stress and negatively regulates *IGFBP4.* As vascular pathology progresses, with increasing stress and the establishment of a complex microenvironment within the plaque, the finely-tuned system dominated by *KLF15* begins to break down, diminishing its negative regulatory effect. This leads to an increase in *IGFBP4* expression, promoting the transformation of these cells into fibroblast-like smooth muscle cells. These cells secrete higher levels of collagen and extracellular matrix components to maintain plaque stability.

Nevertheless, we acknowledge several limitations in our study. First, our research is primarily based on the analysis of publicly available data. Due to the limited number of samples and the absence of survival information, our study cannot be directly associated with clinical prognosis. Second, the absence of experimental animals with specific vascular smooth muscle cell knockout capabilities to validate the results of our bioinformatics analysis and elucidate specific molecular mechanisms represents a limitation.

## Conclusion

Our study not only utilized multi-omics analysis and machine learning algorithms to establish a plaque diagnostic and assessment model based on smooth muscle cell subpopulations but also delved into the molecular interaction mechanisms of smooth muscle cells within atherosclerotic plaques. This provides a fundamental understanding for innovative diagnostic and therapeutic approaches. Further prediction analysis of smooth muscle cell-specific transcription factors revealed that the transcription factor *KLF15* may regulate the PI3K-AKT signaling pathway through the *KLF15/IGFBP4* axis, modulating smooth muscle cells and ultimately affecting the biological behaviors of smooth muscle cells, thus influencing the stability of advanced plaques. This emerging focus highlights its potential as a therapeutic target, driving advancements in the field and bringing us closer to more effective strategies for managing and treating atherosclerosis.

### Electronic supplementary material

Below is the link to the electronic supplementary material.

Supplementary Material 1

## Data Availability

The datasets analysed during the current study are available in GEO datasets. GSE159677 (https://www.ncbi.nlm.nih.gov/geo/query/acc.cgi?acc=GSE159677),GSE28829 (https://www.ncbi.nlm.nih.gov/geo/query/acc.cgi?acc=GSE28829),GSE43292 (https://www.ncbi.nlm.nih.gov/geo/query/acc.cgi?acc=GSE43292),GSE100927 (https://www.ncbi.nlm.nih.gov/geo/query/acc.cgi?acc=GSE100927), and GSE20129 (https://www.ncbi.nlm.nih.gov/geo/query/acc.cgi?acc=GSE20129).

## Abbreviations

VSMCs: Vascular smooth muscle cells
GEO: Gene expression omnibus
WGCNA: Weighted gene co-expression network analysis
LASSO: Least absolute shrinkage and selection operator
RF: Random forest
RT-qPCR: Reverse-transcription quantitative-polymerase chain reaction
KLF15: Krüppel-like family 15
AS: Atherosclerosis
MI: Myocardial infarction
ECM: Extracellular matrix
IGFBP4: Insulin like growth factor binding protein 4
APOE: Apolipoprotein E
CTSC: Cathepsin C
AC: Aherosclerotic core
PA: Proximal adjacent
DEGs: Differentially expressed genes
TOM: Topological overlap matrix
MM: Module membership
GO: Gene ontology
KEGG: Kyoto encyclopedia of genes and genomes
GSEA: Gene set enrichment analysis
AUC: Area under the curve
SD: Sprague-dawley
H&E: Hematoxylin eosin
SDS-PAGE: Sodium dodecyl sulfate-polyacrylamide gel electrophoresis
TBST: Tris-buffered saline and Tween-20
PVDF: Polyvinylidene fluoride
HRP: Horseradish peroxidase
ECL: Enhanced chemiluminescence
ANOVA: Analysis of variance
BP: Biological processes
CC: Cellular components
MF: Molecular functions
DCA: Decision curve analysis
WB: Western blot
TFs: Transcription factors
PCA: Principal component analysis
