# Supplementary figures and images for: Deciphering smooth muscle cell heterogeneity in atherosclerotic plaques and constructing model: a multi-omics approach with focus on KLF15/IGFBP4 axis
Source: BMC Genomics. 2024 May 17;25:490. doi: 10.1186/s12864-024-10379-y (PMC11102212; doi:10.1186/s12864-024-10379-y)

Control   Early   Advanced

35kDa —  
25kDa —

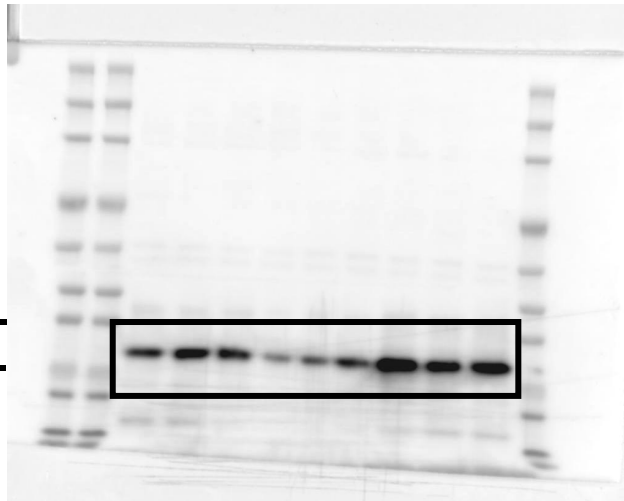

Igfbp4

70kDa —  
50kDa —  
40kDa —

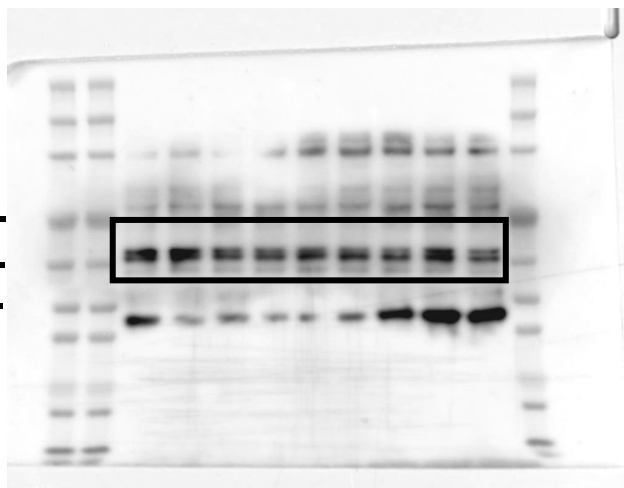

$\alpha$ -Tubulin

Supplement: Supplementary file 1 — Supplementary Material 1 [file 12864_2024_10379_MOESM1_ESM.pdf]
